# Train Distance Estimation in Turnout Area Based on Monocular Vision

**DOI:** 10.3390/s23218778

**Published:** 2023-10-27

**Authors:** Yang Hao, Tao Tang, Chunhai Gao

**Affiliations:** 1 School of Electronic and Information Engineering, Beijing Jiaotong University, Beijing 100044, China; ttang@bjtu.edu.cn; 2 Traffic Control Technology Co., Ltd., Beijing 100070, China; chunhai.gao@bj-tct.com

**Keywords:** autonomous driving, urban railway transit, object detection, vision, instance segmentation

## Abstract

Train distance estimation in a turnout area is an important task for the autonomous driving of urban railway transit, since this function can assist trains in sensing the positions of other trains within the turnout area and prevent potential collision accidents. However, because of large incident angles on object surfaces and far distances, Lidar or stereo vision cannot provide satisfactory precision for such scenarios. In this paper, we propose a method for train distance estimation in a turnout area based on monocular vision: firstly, the side windows of trains in turnout areas are detected by instance segmentation based on YOLOv8; secondly, the vertical directions, the upper edges and lower edges of side windows of the train are extracted by feature extraction; finally, the distance to the target train is calculated with an appropriated pinhole camera model. The proposed method is validated by practical data captured from Hong Kong Metro Tsuen Wan Line. A dataset of 2477 images is built to train the instance segmentation neural network, and the network is able to attain an MIoU of 92.43% and a MPA of 97.47% for segmentation. The accuracy of train distance estimation is then evaluated in four typical turnout area scenarios with ground truth data from on-board Lidar. The experiment results indicate that the proposed method achieves a mean RMSE of 0.9523 m for train distance estimation in four typical turnout area scenarios, which is sufficient for determining the occupancy of crossover in turnout areas.

## 1. Introduction

In recent years, the development of visual sensors and detection algorithms has brought new opportunities for the autonomous driving of urban rail transit trains [1,2,3]. By leveraging sensors such as cameras, Lidar (Light detection and ranging), millimeter-wave radar, and infrared cameras, trains can accomplish tasks such as object detection [4], distance measurement [5], track area inspection [6], and autonomous train positioning [7], partially replacing the role of train drivers. However, for some tasks that involve complex scenarios in urban rail transit, the performance of current technology is still insufficient. Train distance estimation in turnout areas, for instance, stands out as one of the most challenging tasks.

**Figure 1:**
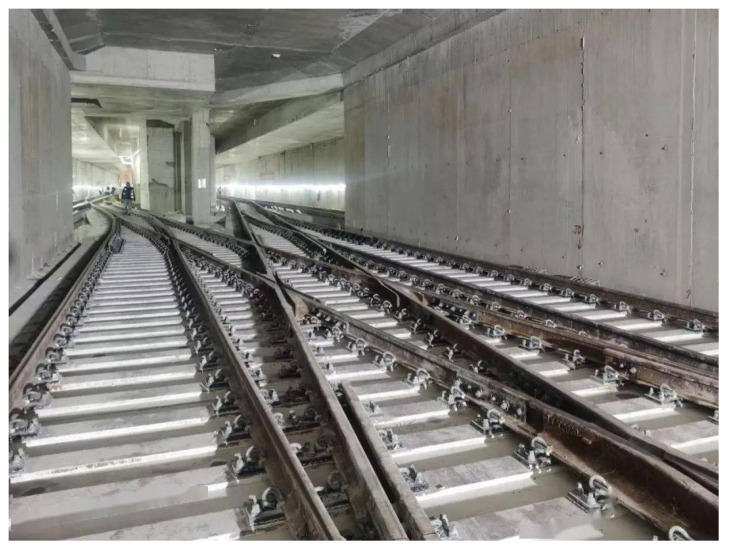
A typical turnout area in urban rail transit.

Turnout is a vital component of railway systems, mainly positioned in areas such as yards and terminal loops [8], where trains need to be switched from one track to another through crossovers, as shown in Figure 1. For trains utilizing traditional signal systems, they can be navigated safely through turnouts into the next section under the protection of the signal system’s interlocking mechanism [9]. However, in cases where a train cannot access the signal system, it has to accurately determine whether the turnout ahead is occupied by other vehicles to prevent collision. To accurately determine the turnout occupancy, one of the most crucial tasks is the distance measurement of trains on adjacent or opposing tracks, since it further influences the train’s operation plan, which has to balance the operational efficiency and safety.

**Figure 2:**
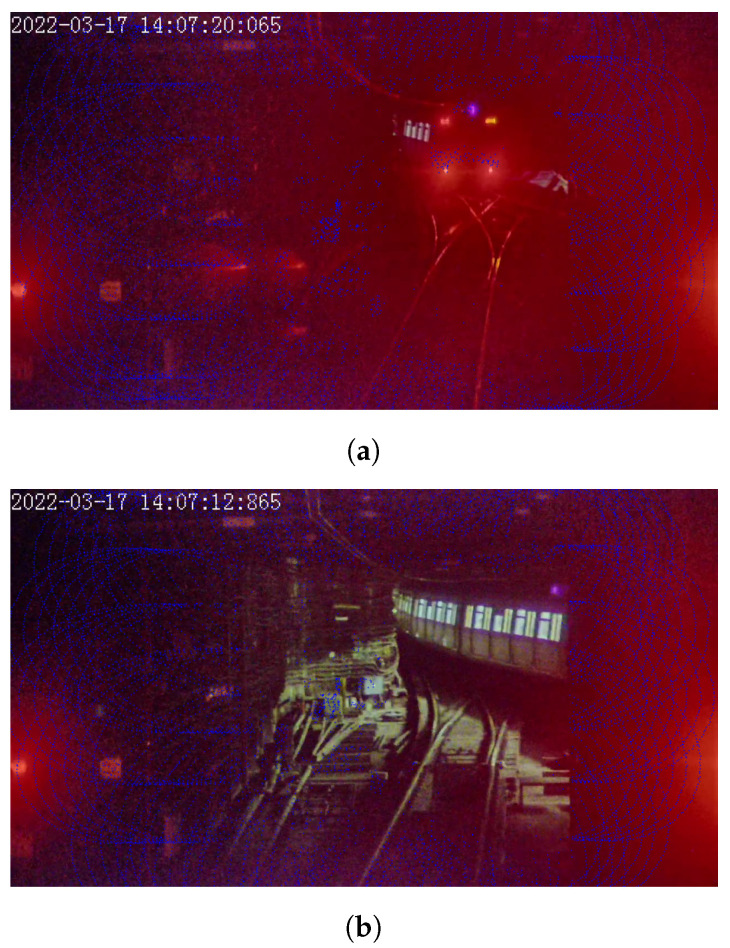
Point clouds on train in turnout area (plotted by blue dots). (**a**) Returned point clouds on train head. (**b**) No point clouds on train body.

For distance estimation, Lidar is generally considered to be the most suitable sensor for its far measuring range and high accuracy. The basic theory of Lidar is to calculate the distance to objects by emitting and receiving laser pulses and measuring their return time. Due to its high precision and extended range [10], Lidar is widely used in applications such as precise positioning, obstacle avoidance, map creation, and autonomous driving [11,12]. However, for distance estimation in turnout areas of urban rail transit, the train body cannot be detected robustly by on-board Lidar, and this poses significant threats to operation safety. As shown in Figure 2, when we overlay the synchronized images and point clouds captured from camera and Lidar together, we can see the train head in Figure 2a shows a lot of returned point clouds (as plotted by blue dots), while the train body in Figure 2b shows no returned point cloud. The reason behind this is that laser pulses emitted by the Lidar experience severe energy attenuation in cases where the incidence angle on the object surface is too large [13]. When the echo energy falls below the detection threshold, the Lidar will fail to detect the target object [14]. Based on the above-mentioned reason, Lidar is not robust for distance estimation in turnout areas.

Apart from Lidar, camera vision is another suitable choice for distance measurement in the field of autonomous driving. While cameras cannot directly acquire distance information like Lidar, they indirectly acquire distance information by distance estimation algorithms. Unlike the active ranging of Lidar which involves emitting and receiving laser pulses, cameras passively capture natural light reflected from the detected objects’ surfaces, thus they can reliably acquire information of color, texture, and light intensity of targets [15]. While camera-based distance measurement may not achieve the precision of Lidar, the richness of its image information enables the distance estimation task in tasks, which Lidar cannot achieve.

In most cases, object distance estimation in computer vision is achieved by using stereo vision, and the depths are estimated by triangulation of feature points. which are extracted and matched from two stereo images [16]. For short-range distance estimation applications, stereo vision provides satisfactory results in spite of several usual shortcomings such as unreliable stereo correspondence solution in textureless image regions [17]. However, distance estimation based on stereo vision is characterized with inaccuracy in estimation of larger distances [18]. For the on-board cameras on urban rail transit trains, the baseline of stereo vision can not exceed the width of the train head, i.e., less than 3 m, while the distance estimation range is always more than 30 m or even much farther as 100 m, therefore the accuracy of distance estimation is not sufficient.

In order to overcome the problems of stereo vision in the estimation of relatively far distances, a number of authors have proposed solutions based on monocular vision. Unlike stereo vision, monocular distance measurement relies solely on images captured by a single camera, the relative distance of objects is inferred by analyzing the information of object size, shape, perspective relationships, and combining this with the camera’s intrinsic and extrinsic parameters. This approach often requires apriori environmental information or camera calibration to provide more accurate distance measurement results. For far distance estimation in rail transit application, Ref. [19] proposes a homography-based method to estimate the distances from the camera stand to objects and pedestrians on railway tracks. Ref. [20] proposes a method that solves a Perspective-2-Point problem to estimate the distance between the locomotive and the wagons. However, these methods are based on the hypothesis that the camera and the objects are on the same rail track, and the rail track needs to be straight, thus the geometric relations between the camera and the rail track can be calculated. To overcome the limitation of traditional vision methods, deep-learning-based object detection has also been considered by researchers to solve monocular distance estimation problems. Ref. [21] proposes an artificial neural network named DisNet for monocular distance estimation of human beings and cars, the objects are detected with bounding box and the neural network is trained to mapping the targets’ pixel sizes and their distances to the camera. Though this method provides distance estimation results in wider range of scenarios, it still has two significant limitations: firstly, the bounding box only provides a rough estimation of the target size, thus its accuracy in scale calculation is limited; secondly, the bounding-box-based method assumes that the target is directly facing the camera, and it performs poorly due to its lack of descriptive capabilities when the target is at different view angles to the camera [22].

Comparing to detection with bounding box, instance segmentation provides pixel-wise contour of the detected object, therefore improves the accuracy of target size estimation [23,24]. Moreover, the contours of segmentation provide more geometric features of detected objects, which can be further extracted as constraints for distance estimation. Therefore, it is reasonable to utilize instance segmentation to extract objects of certain scale on the target train body to obtain more accurate estimation of distance with monocular vision. Based on the above-mentioned analysis and consideration, in this paper, we propose a monocular vision based train distance estimation method for occupancy detection of turnout aresa in urban rail transit autonomous driving. The main contributions of this paper are three-fold:

(1) A monocular vision-based distance estimation framework is designed to achieve the detection of trains in turnout areas. According to the authors’ knowledge, this problem has not been solved with other sensors or strategies, such as Lidar and stereo vision.
(2) An instance segmentation strategy is proposed for train side window detection. By treating multiple side windows in each train carriage as separate entities, the segmentation can be achieved robustly and accurately even in poor illuminance conditions.
(3) A geometric feature extraction strategy is proposed to obtain the quantitative representation of train side window contours, thus the further scale-based distance estimation could be achieved with an acceptable accuracy in practical usage.

The remainder of this paper is structured as follows: Section 2 presents the proposed train distance estimation framework. Section 3 shows the experiment results and discussion. Finally, a conclusion is presented in Section 4.

## 2. Method

### 2.1. Framework Overview

**Figure 3:**
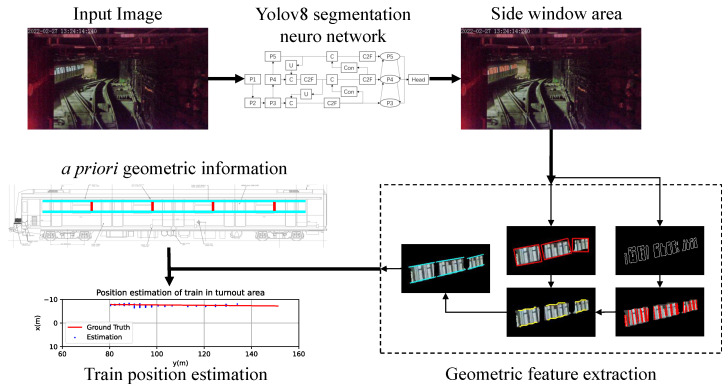
Framework of the proposed train distance estimation method.

In this paper, we propose a method of distance estimation of trains in turnout areas based on monocular vision. The main idea is to extract features of known dimensions by instance segmentation, and estimate the distance to the target train with an approximated pinhole camera model, as shown in Figure 3.

The proposed method consists of two main parts: instance segmentation of train side windows and distance estimation based on geometric features.

### 2.2. Instance Segmentation of Train Side Windows

#### 2.2.1. Train Side Window Segmentation Strategy

**Figure 4:**
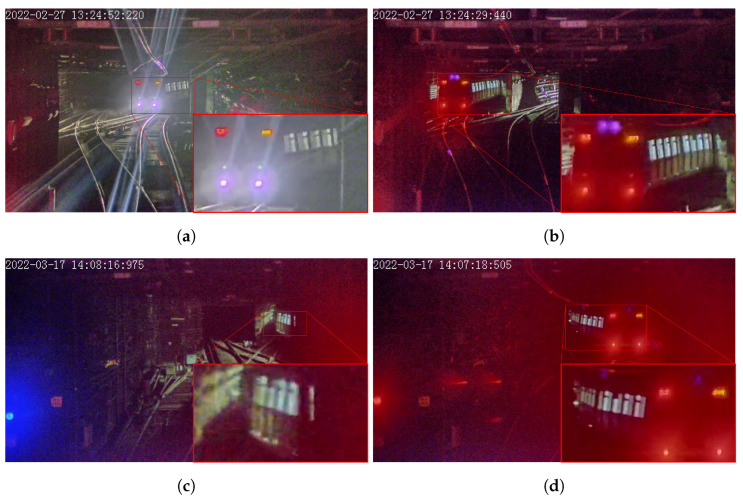
Typical side windows of target train. (**a**) Side windows blurred by train’s head light. (**b**) Side windows captured with large incident angle. (**c**) Side windows captured with far distance. (**d**) Side windows captured with poor illumination. (The red radiance is caused by the tail light of the target train).

Visible light cameras are sensitive to environmental light interference. To adapt scenarios with different illuminance conditions in urban rail transit, especially for those in tunnels with no extra illumination, in this paper, we select the side window of the train as the object to calculate the scale factor of distance estimation. The main reasons are two-fold. Firstly, the side window of the train has a rectangular shape, and the width and height of the windows are constant values. Secondly, the side window of train remains bright even in tunnels with no extra illuminance, thus it can always be detected according to the contrast with the dark background, as shown in Figure 4. Although the train body itself also provides regular geometric features, since its edges are always blurred due to insufficient illuminance, they are not robust for camera-vision-based detection and segmentation.

**Figure 5:**
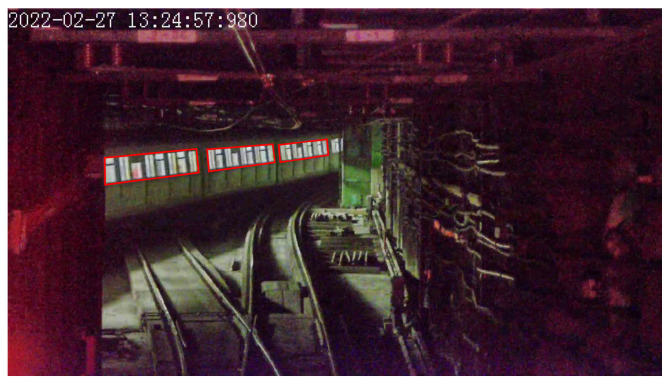
Side windows in the same train carriage (depicted with red boxes).

The segmentation of an independent side window is not an easy task, since the contour of an independent window is not clear when it is captured from a far distance, as shown in Figure 4c. Furthermore, because the independent window lacks texture, the segmentation network always predicts false positives of other objects that have a similar color to the window. Based on the consideration above, we choose the 9 side windows in each train carriage as a single entity, as depicted in red color in Figure 5, since they always align in a vertical direction and create stable and unique textures, which are easier to detect. Even in a very far distance, which makes the image of windows blurred, it still provides sufficient texture to be detected and does not lead to a false positive.

#### 2.2.2. Instance Segmentation with YOLOv8

**Figure 6:**
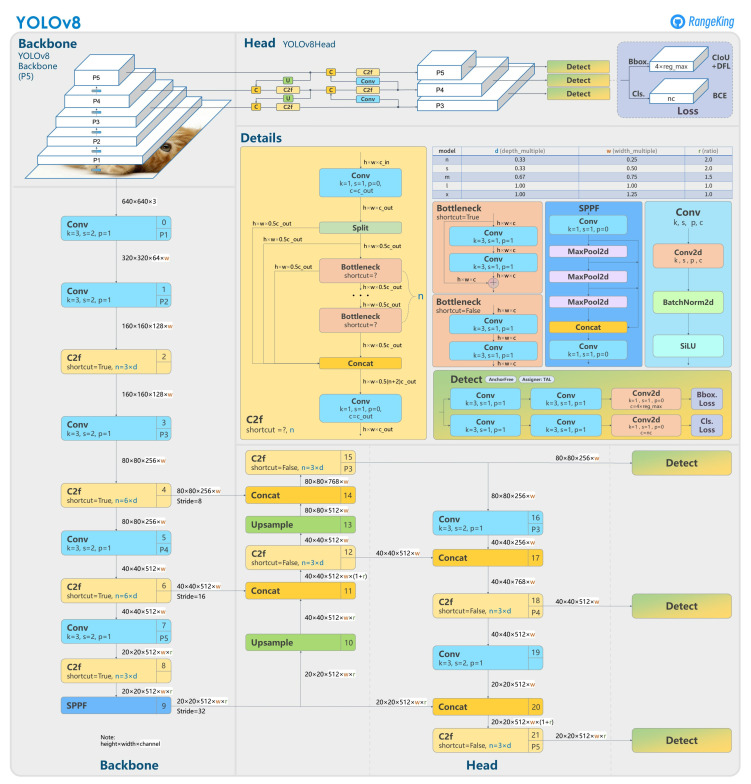
Structure of YOLOv8 network.

Instance segmentation goes beyond semantic segmentation by not only assigning each pixel a semantic label, but also distinguishing the boundaries of individual object instances. It provides unique identifiers for each instance of the same class, accurately outlining the contours of each object and highlighting their independent presence in the image. This is useful when more than one train carriages show in the captured image of on-board camera. In this paper, we choose YOLOv8 as the neural network model for instance segmentation. YOLO (You Only Look Once) is a popular object detection and image segmentation model, which was developed by Joseph Redmon and Ali Farhadi at the University of Washington [25]. As a cutting-edge, state-of-the-art (SOTA) model, YOLOv8 builds on the success of previous versions, introducing new features and improvements for enhanced performance, flexibility, and efficiency [26]. YOLOv8 supports a full range of vision AI tasks, including detection, segmentation, pose estimation, tracking, and classification. This versatility allows users to leverage YOLOv8’s capabilities across diverse applications and domains. The main structure of the YOLOv8 network is as shown in Figure 6.

After training with the appropriate dataset, the model can provide pixel-wise regions of side windows of target trains.

### 2.3. Distance Estimation Based on Geometric Features

With the segmented regions of side window, the geometric features should be extracted to estimate the scale factor. Since the side windows of train have rectangular shape, their width and height both provide scale information in monocular distance estimation, theoretically. However, due to potential obstructions in the train operating environment, such as tunnels walls, it is quite difficult to detect the entire lateral length of a train carriage’s window in a randomly given frame of image. Therefore, the horizontal dimension of the window is not an appropriate geometric feature for distance estimation. On the other hand, the vertical dimension of the window is less susceptible to obstruction and is better suited as a geometric feature for distance estimation.

**Figure 7:**
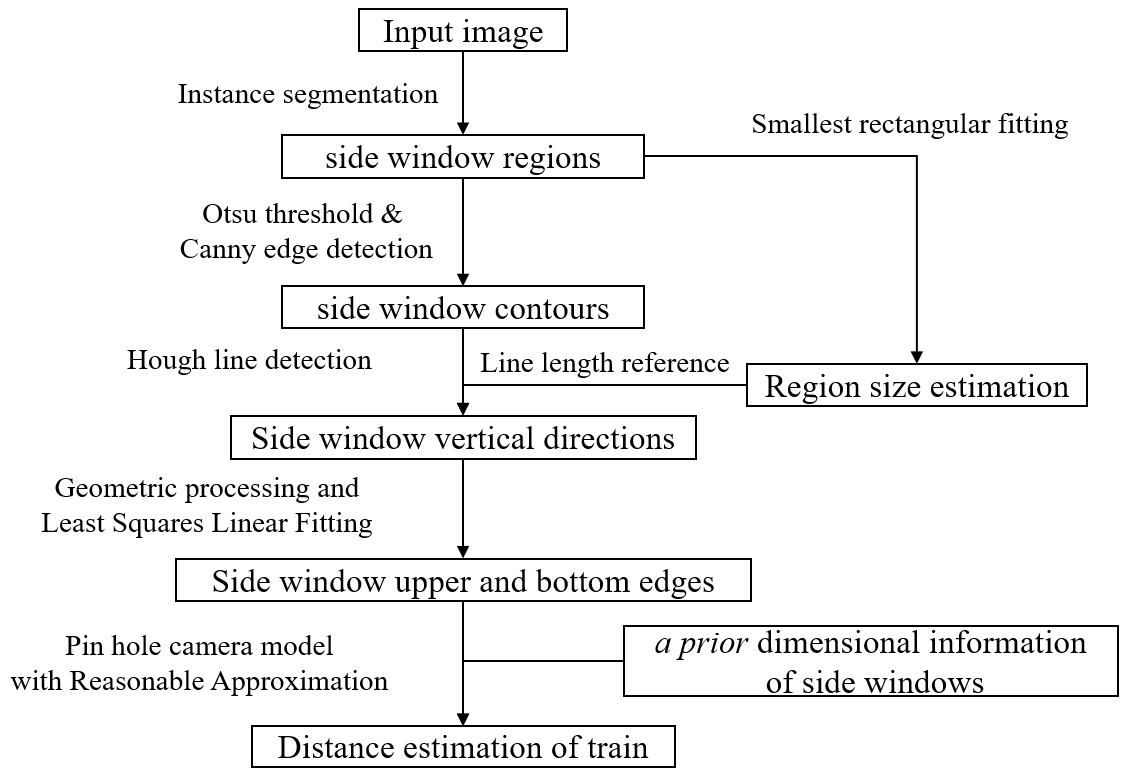
Workflow of distance estimation.

Therefore, in this paper, we choose the vertical height of side windows as the key to calculate the scale factor and estimate the distance to the target train. The workflow after instance segmentation is as shown in Figure 7.

#### 2.3.1. Vertical Directions of Side Windows

To obtain the pixel height of the side window in the image, the vertical direction of the side window is needed. We here calculate the vertical direction in 3 steps.

**Figure 8:**
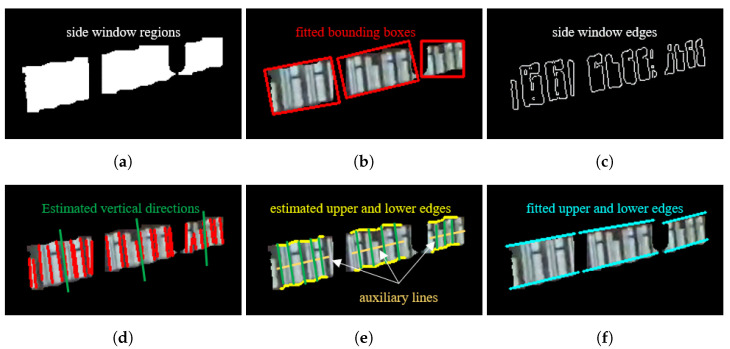
Main steps of geometric feature extraction of train side windows. (**a**) Side window regions obtained by instance segmentation. (**b**) Minimum bounding box fitting for side windows. (**c**) Side window edges obtained by Otsu’s thresholding and Canny edge detection. (**d**) Estimated vertical directions of side windows (the red lines are the vertical lines detected by Hough line detection, and the green lines are their averages). (**e**) Auxiliary lines (khaki) and detected upper and lower edges of side windows (yellow). (**f**) Fitted upper and lower edges of side windows (blue).

(1) By performing minimum bounding box fitting on the instance segmentation regions, a rough estimation of the width and height of the windows is obtained, as depicted with red rectangles in Figure 8a,b.
(2) By locally applying Otsu’s thresholding and Canny edge detection in the instance segmentation regions, the contour edges of individual side windows within each train carriage are obtained, as shown in Figure 8c.
(3) By employing Hough line detection to the contour edges, a bunch of straight lines can be extracted. Considering the dimensions and main directions of the fitted bounding boxes, the lines for vertical edges of side windows are then determined, and by computing the directional average of the detected vertical lines, the optimal estimation for the vertical dimension of the side windows is obtained, as depicted with green lines in Figure 8d.

#### 2.3.2. Upper and Lower Edges of Side Windows

To estimate the distance of the whole target train side window, upper and lower edges of side windows are needed. However, the edges of the side window areas detected by instance segmentation are often curved rather than straight lines, which may cause errors for distance estimation. Therefore, to obtain well-aligned upper and lower edges of side windows, we here use linear fitting to solve this problem in 3 steps.

(1) At the center of the minimum bounding box obtained from instance segmentation, auxiliary lines are constructed perpendicular to the side window’s vertical direction. Along the auxiliary lines, multiple sampling points are selected at a certain interval, as depicted with the khaki color in Figure 8e.
(2) Starting from these sampling points, extension lines are drawn in both the positive and negative directions along the side windows’ vertical directions. The intersection points of these extension lines with the instance segmentation contours are then calculated as the estimation for the upper and lower edges of the side window, as depicted with a yellow color in Figure 8e.
(3) Linear fitting is separately applied to the sets of points representing the upper and lower edges, resulting in linear estimation for the upper and lower edges of the side window, as depicted with blue lines in Figure 8e.

#### 2.3.3. Distance Estimation Based on Approximated Pinhole Imaging Model

The basic idea of the monocular distance estimation is to estimate the scale factor from apriori information of the object. In this paper, the distance from the camera to the target train is supposed to be a value much larger than the height of side window, therefore some approximations can be made to simplify the calculation while still keeping the accuracy.

**Figure 9:**
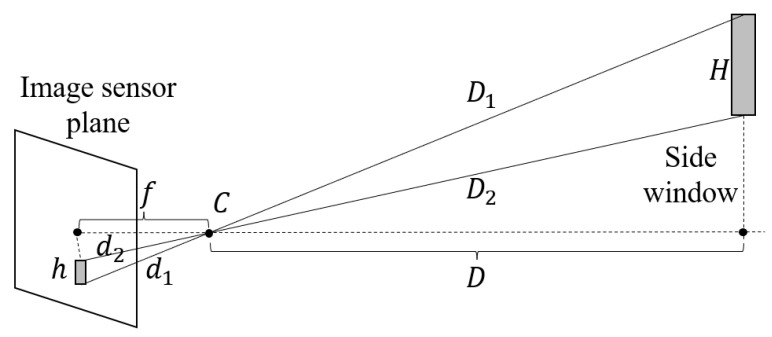
Approximated pinhole camera model for far distance estimation.

As shown in Figure 9, if we suppose that the on-board camera is a pinhole camera with a focal length of *f*, for a piece of train side window with a height of *H*, it can be projected to the image sensor plane with a pixel height of *h*. We denote the distances from the upper and lower edges to the camera optical center *C* as $D_{1}$ and $D_{2}$, and the distances from the edges in its image as $d_{1}$ and $d_{2}$. If there is no significant altitude gap between the on-board camera and the side window, and *D* is significantly larger than *H*, we can obtain an approximation that $D\approx D_{1}\approx D_{2}$. And if the on-board camera uses a telephoto lens, we can further obtain another approximation that $f\approx d_{1}\approx d_{2}$. Based on the above-mentioned approximations, since we are only concerned about the distance *D* in depth direction and do not care about altitude direction, we can simply calculate the distance by using similar triangles, as Equation (1)

(1) $$D=H\frac{f}{h}$$

## 3. Experiments

To validate the effectiveness of the proposed method and evaluate its performance, we implemented experiments in the turnout area of the Hong Kong Metro Tsuen Wan Line.

### 3.1. Experiment Setup

**Figure 10:**
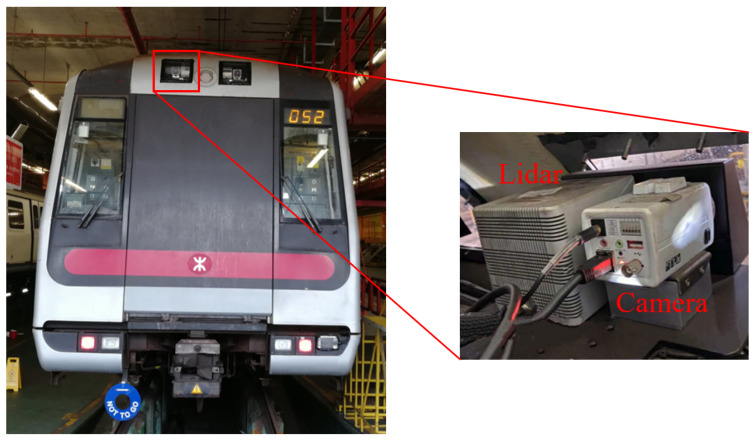
Sensor setup in Hong Kong Metro Tsuen Wan Line.

As shown in Figure 10, a telephoto camera and a Lidar are mounted inside the roof of the train by means of a fixed bracket. The camera in the setup is a custom-designed camera with built-in high dynamic range (HDR) capability, thus enables starlight-level imaging and has a pixel resolution of 1280 × 720. Equipped with a 25 mm telephoto lens, the camera offers a field of view of 13${}^{\circ }$ in horizontal direction and 14${}^{\circ }$ in vertical direction. The Lidar used in this study is a Livox Tele-15 [27] with a field angle view of 14.5${}^{\circ }$× 16.2${}^{\circ }$. To get a better forward view, the camera’s pitch Angle is set to 8 degrees, while the Lidar is mounted horizontally.

**Figure 11:**
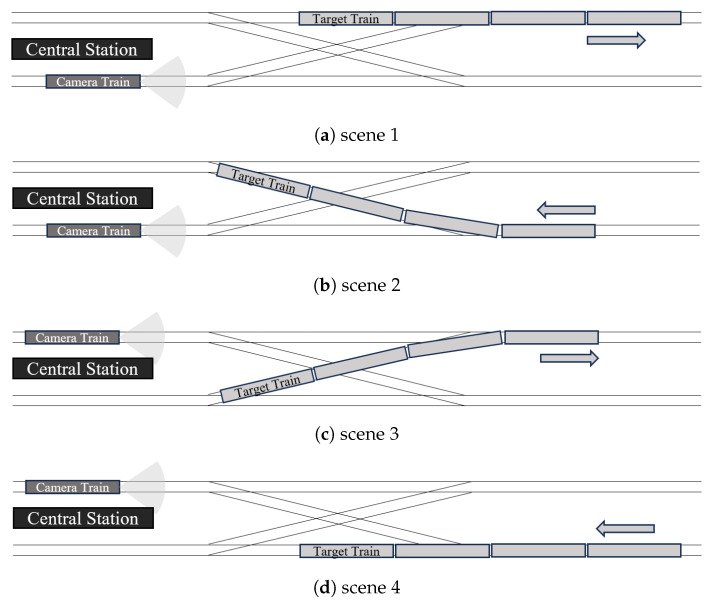
Typical scenes in turnout area. The arrows depicted in the sub figures are to show the moving directions of the target trains.

In the experiments of this study, we choose four typical scenarios in the turnout area in Central Station of Hong Hong Metro Tsuen Wan Line, as shown in Figure 11. In these scenarios, the train proceed into the turnout area in an upward direction, and then move onto the downward track. Due to the tight scheduling of train services, one train in the downward direction must wait until the other leaves the crossover for safety.

### 3.2. Instance Segmentation

#### 3.2.1. Dataset and Model Training

**Figure 12:**
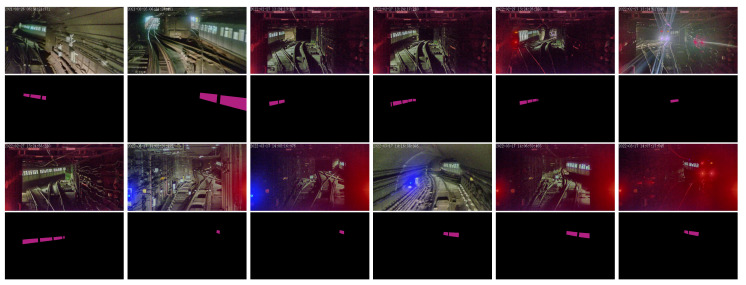
Typical images of dataset. The purple regions are the masks for side window segmentation training.

To build the dataset for neural network training, we diligently annotated a total of 2477 images with more than 7000 train side window instance segmentation labels, as shown in Figure 12. The dataset was separated into training, validation, and test sets with a distribution ratio of 6:2:2.

YOLOv8 provides five pre-trained models of different sizes for the instance segmentation task. In this paper, to obtain the balance between efficiency and speed, we chose the “YOLOv8m-seg” model. The input resolution of this model is 640 pixels, and has 27.3 M parameters (110.2B FLOPs).

The network training was performed utilizing an Nvidia RTX3070 GPU. Taking into consideration the 8 GB VRAM and the size of the model, we configured the batch size as 8 for 500 epochs. Other detailed information of the training configuration is as shown in Table 1.

To enhance the network’s adaptability, we also employed data augmentation during the training process. This involved adjustments to the original dataset such as HSV (Hue, Saturation, Value) adjustments, translation range, scale range, etc. The details of data augmentation are as shown in Table 2.

**Figure 13:**
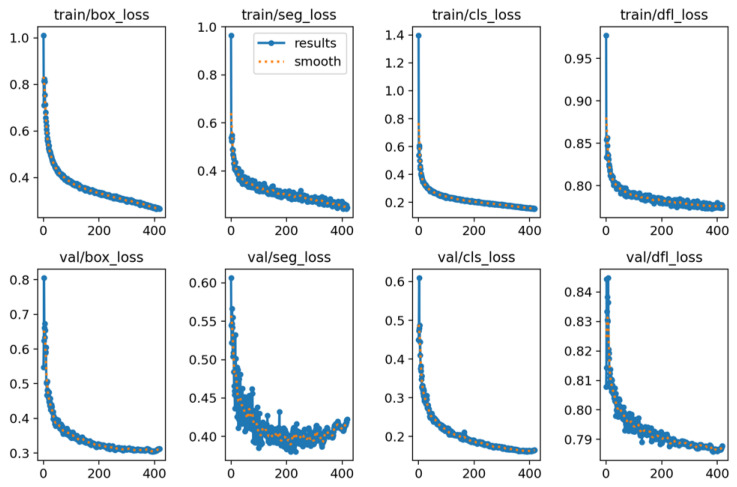
The tendency of losses during training.

**Figure 14:**
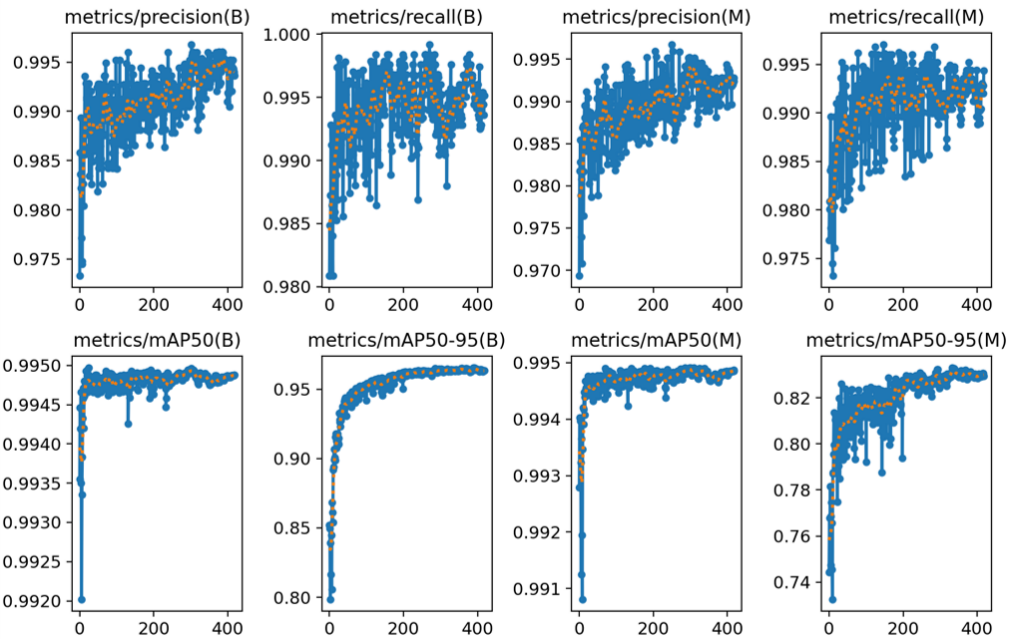
The tendency of precision, recall and mAPs during training. The blue lines show the original values of the parameters, while the orange dots show the smoothed values.

The tendency of losses, precision, recall and mAPs are as shown in Figure 13 and Figure 14. As the loss did not decrease after the 369th epoch, the train process automatically stopped at the 420th epoch with a patience of 50.

#### 3.2.2. Instance Segmentation Performance

**Figure 15:**
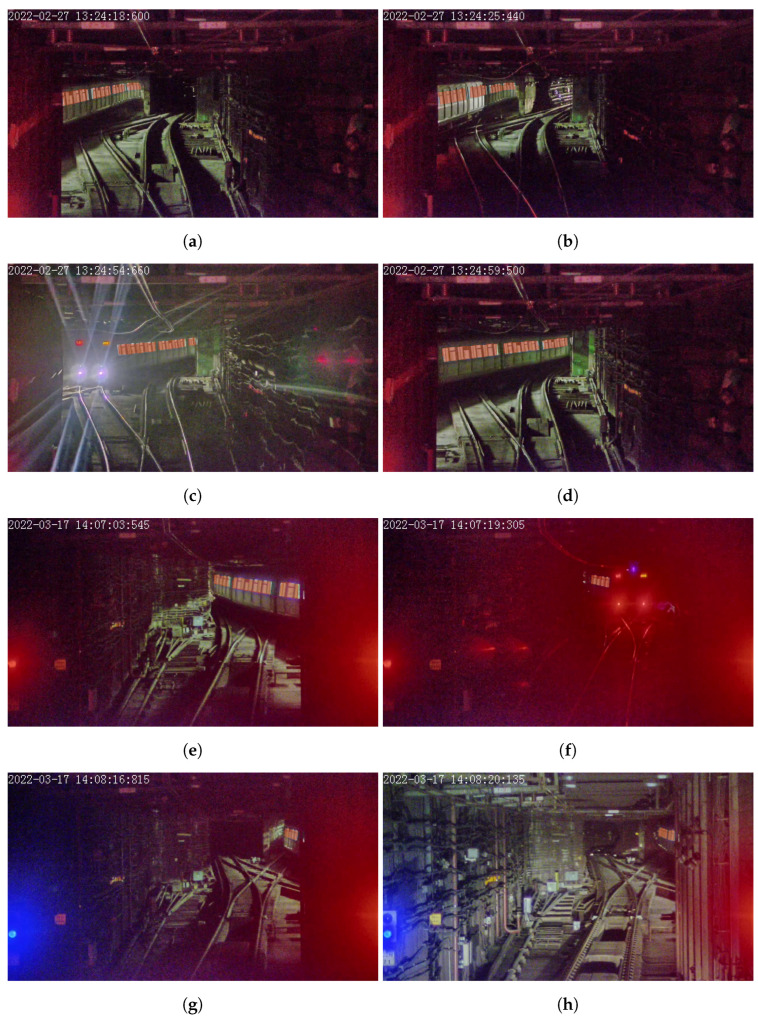
Results of train side window segmentation. (**a**) Segmented side windows in scene 1. (**b**) Segmented side windows in scene 1, when the target train body was illuminated by other train’s head light. (**c**) Segmented side windows in scene 2, when blurred by white head light. (**d**) Segmented side windows in scene 2. (**e**) Segmented side windows in scene 3. (**f**) Segmented side windows in scene 3, when blurred by red tail light. (**g**) Segmented side windows in scene 4, when camera train’s head light is off. (**h**) Segmented side windows in scene 4, when camera train’s head light is on.

For the train side windows in the four typical scenarios, the segmentation results are as shown in Figure 15, and the segmented regions are depicted with transparent red color.

For scene 1, the results in Figure 15a,b show that even when the target train is illuminated by the headlights of other trains from the other side, the side windows of the target train can still be detected robustly.

For scene 2, the results in Figure 15c,d show that even when the headlights of the target train are extremely bright, causing a reduction in camera exposure, the side windows of the target train can still be detected reliably.

For scene 3, the results in Figure 15e,f show that as long as the windows of the target train are visible, it can still be detected even when the train body is not visible.

For scene 4, the results in Figure 15g,h show that even there is only one carriage of the target train is available, the side window of the can still be detected, regardless of interference of external illumination.

For further quantitative evaluation of instance segmentation performance, two popular indicators, the Mean Intersection-over-Union (MIoU) metric and the mean pixel accuracy (MPA) metric were used in this paper, as shown in Equations (2) and (3),

(2) $$MIoU=\frac{1}{n+1}\sum_{i=0}^{n}\frac{TP_{i}}{TP_{i}+FP_{i}+FN_{i}}$$

(3) $$MPA=\frac{1}{n+1}\sum_{i=0}^{n}\frac{TP_{i}}{TP_{i}+FN_{i}}$$

where n + 1 represents the number of segmentation categories (n + 1 = 2 in this study); $TP_{i}$ refers to true positive pixels about category *i*, and $FN_{i}$ and $FP_{i}$ refer to false negative pixels and false positive pixels about category *i*, respectively. Based on the results from the test set, the instance segmentation network model trained by us achieved a MIoU of 92.43% and a MPA of 97.47%.

The above-mentioned results show that the proposed side window segmentation strategy works with an acceptable accuracy even in scenarios of bad illumination conditions. Although the segmented contours of the side windows are not as smooth as the annotation, they still keep regular shapes and dimensions, which can provide sufficient geometric information for the subsequent process of scale calculation.

### 3.3. Target Train Distance Estimation

#### 3.3.1. Ground Truth Acquisition

**Figure 16:**
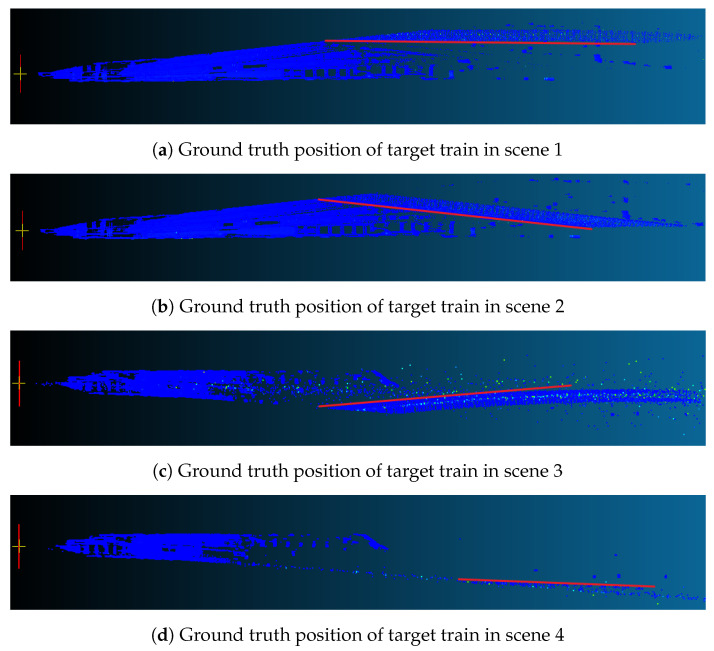
Ground truth positions of train body in 4 typical scenes. The yellow crosses are the origins under Lidar reference frame, the blue dots are the overlayed point clouds, and the red straight lines are the fitted truth positions of train body.

For performance evaluation of train distance estimation, ground truth distance to the target train is the best choice. Even though the train body cannot provide a returned point cloud due to large incidence angles on object surfaces, there are other cases in which the train head provides returned point clouds. By this consideration, we overlay the point cloud frames in which the train head or tail is still detectable and thus obtained the ground truth position of the train body in the four scenes, as shown in Figure 16. For ease of further evaluation of distance estimation, we use the least square linear fitting to describe the ground truth edge of the target train body, as indicated by the red lines depicted in the figure.

**Figure 17:**
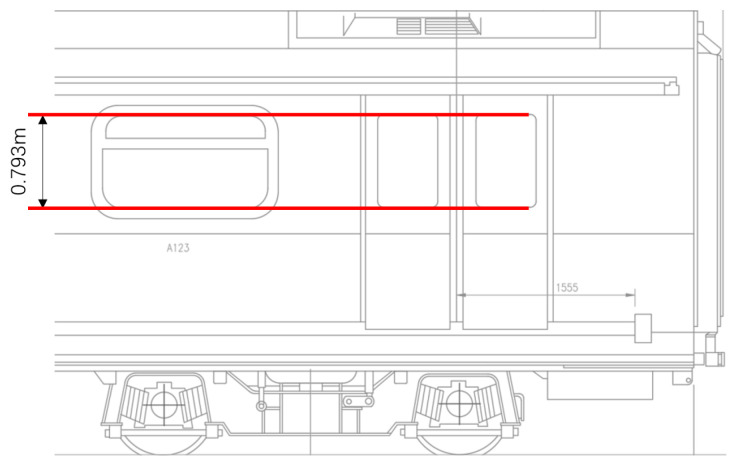
Side window height measured in Hong Kong Metro Tsuen Wan Line.

For apriori dimensional information, the side window height value is obtained from the carriage script of the Hong Kong Metro Tsuen Wan Line. As shown in Figure 17, the height of the side windows is 0.793 m.

#### 3.3.2. Distance Estimation Performance

**Figure 18:**
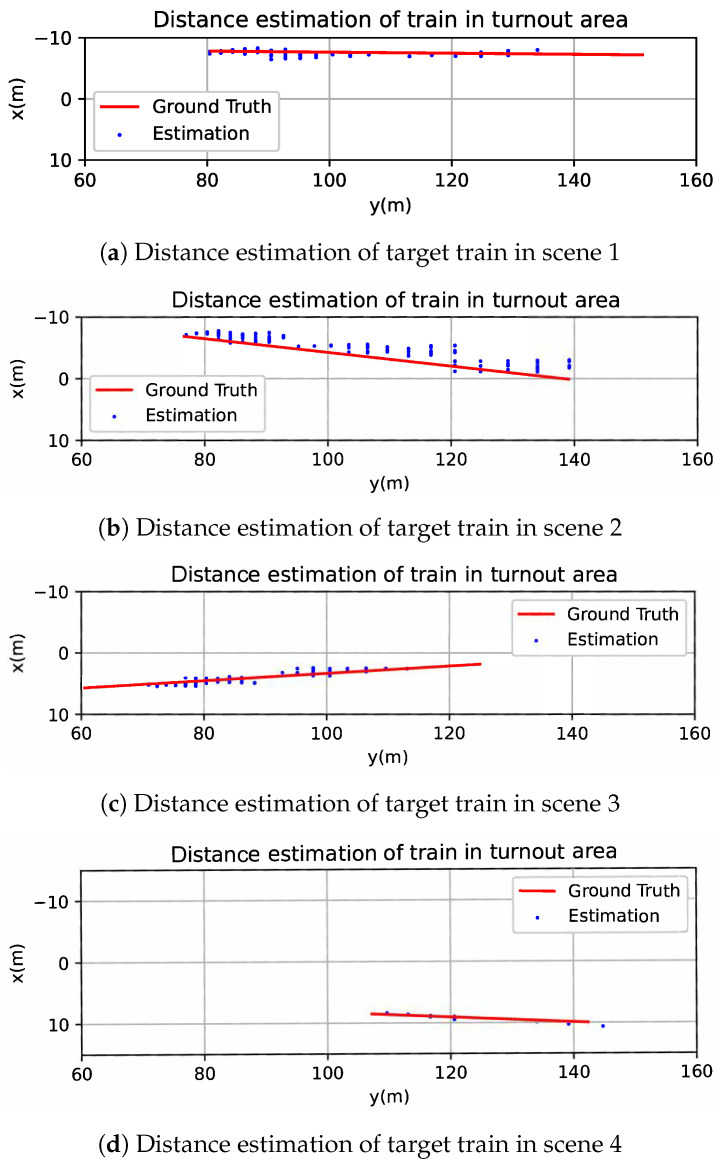
Distance estimation of train side windows with proposed method.

Based on the above-mentioned experiment configuration and ground truth values, distance estimation experiments were implemented. To maintain generality, we selected sampling points at a one pixel interval within the window area and calculated distances by combining the corresponding window pixel height at each sampling point. The results of train position estimation of the four typical scenes are as shown in Figure 18.

For quantitative evaluation, we use RMSE (root mean square error) to evaluate the error of distance estimation of train side windows, as shown in Table 3. From the results, we can know that the proposed method performs better in scene 1 and scene 4, where the target trains are parallel to the camera train.

Since the original intention of distance estimation of the target train is to determine the occupancy of crossover, it should be accurate enough to distinguish whether the target train is on a crossover. Since the mean RMSE of distance estimation in four typical scenarios is 0.9523 m and less than the standard gauge of 1.435 m, the proposed train distance estimation framework is considered to be accurate enough for determining crossover occupancy.

## 4. Conclusions

In this paper, we propose a framework of distance estimation of trains in turnout areas based on monocular vision. The side windows of target train are segmented based on YOLOv8 neural network, then the geometric features are extracted for distance estimation. For validation and evaluation, we implement experiments on the Hong Kong Metro Tsuen Wan Line, a dataset that includes 2477 images that are built for instance segmentation model training, and distance estimation experiments are implemented in four typical turnout area scenarios. According to the results, the proposed method can estimate the distance of trains in turnout areas where Lidar fails to obtain a point cloud, and the accuracy is enough for determining crossover occupancy. Furthermore, the proposed method works well in scenes of bad illumination and scenes where only a part of the target train is visible.

However, this paper also has several limitations. Firstly, limited by the quantity of dataset annotation, in this paper we only studied the instance segmentation based on a single image frame. The future work will focus on the enhancement of instance segmentation by exploring a neural network with a temporal context. Secondly, this paper only utilized data from the tunnel section of the Tsuen Wan Line in Hong Kong. The future work will consider data from more urban rail transit lines and scenarios to improve the method’s generalization and robustness. Finally, this paper only utilized side windows as geometric features for scale calculation, future work will also integrate more features to improve the accuracy and robustness of distance estimation.

## Figures and Tables

**Table 1:** Training configuration.

Parameter	Value	Parameter	Value
Epochs	500	Patience	50
Batch	8	Image Size	640
Save	True	Save Period	−1
Device	Auto	Workers	8
Project	Null	Name	Null
Pretrained	True	Optimizer	Auto
Verbose	True	Seed	0
Deterministic	True	Cosine LR	False
Mixed Precision	True	Validation	True
Validation Split	Val	Conf. Threshold	Null
IoU Threshold	0.7	Max Detection	300
Overlap Mask	True	Mask Ratio	4
Dropout	0.0		

**Table 2:** Data augmentation configuration.

Parameter	Value	Parameter	Value
HSV Hue Range	0.015	HSV Saturation Range	0.7
HSV Value Range	0.4	Degrees Range	0.0
Translation Range	0.1	Scale Range	0.5
Shear Range	0.0	Perspective Range	0.0
Vertical Flip Prob.	0.0	Horizontal Flip Prob.	0.5
Mosaic Augmentation	1.0		

**Table 3:** RMSE of target train distance estimation.

Scene	RMSE (Unit: m)
1	0.7020
2	1.0203
3	1.2598
4	0.5239
mean	0.9523

## Data Availability

The data utilized in this study are proprietary and confidential data held by the company Traffic Control Technology Co., Ltd. and are protected by confidentiality agreements and legal regulations. Due to the sensitive and confidential nature of the data, they are not publicly accessible. For further research interests or access requests, please contact the data administrator or relevant department to obtain additional information and permissions.
